# Faricimab efficacy in a poorly responsive case of Polypoidal Choroidal Vasculopathy (PCV): A case report

**DOI:** 10.1177/11206721241310629

**Published:** 2025-02-12

**Authors:** Clara Rizzo, Maria Cristina Savastano, Raphael Kilian, Stanislao Rizzo

**Affiliations:** 1 Ophthalmic Unit, Department of Neurosciences, Biomedicine and Movement Sciences, 9310 University of Verona, Verona, Italy; 2 Ophthalmology Unit, 18654 Fondazione Policlinico Universitario A. Gemelli IRCCS, Rome, Italy; 3 Catholic University of “Sacro Cuore”, Rome, Italy; 4 Department of Traslational Medicine, 9299 University of Ferrara, Ferrara, Italy; 5 Consiglio Nazionale delle Ricerche, Istituto di Neuroscienze, Pisa, Italy

**Keywords:** Faricimab, PCV, PED, anti-VEGF therapy

## Abstract

**Purpose:**

To evaluate anatomical and functional outcomes of Faricimab therapy on a case of polypoidal choroidal vasculopathy (PCV) refractory to previous treatments.

**Major findings:**

A 56-year-old patient with PCV presenting with a large pigment epithelial detachment (PED) showed best-corrected visual acuity (BCVA) improvement, subretinal fluid (SRF) reduction and PED height reduction at 2 months follow up after a loading phase of 4 monthly Faricimab injections. The patient had previously undergone Aflibercept, Brolucizumab and photodynamic therapy (PDT) treatments with a suboptimal response.

**Conclusion:**

Despite Faricimab proving itself as an efficient alternative in a case of PCV poorly responsive to previous treatments, definite evidence still needs to be confirmed in larger studies.

## Introduction

Age-related macular degeneration (AMD) is a leading cause of blindness worldwide and a large portion of the population is estimated to become affected by this disease in the near future.

Polypoidal choroidal vasculopathy (PCV) has been classified as a subtype of neovascular AMD (nAMD). Notably, it has a higher prevalence in asian populations and it tends to have a predominantly hemorrhagic or exudative macular presentation causing significant vision loss and thus greatly invalidating life quality of the patients.

PCV has shown disparity in response to intravitreal injections of anti-vascular endothelial growth factor (VEGF) agents when compared to typical AMD, and additional photodynamic therapy (PDT) and/or laser is often required. Currently, the best treatment for PCV has remained unclear.

In 2022 the FDA approved a new anti-VEGF agent called Faricimab, a bispecific antibody that acts through dual inhibition of both angiopoietin-2 and vascular endothelial growth factor A. Two studies in phase 3 (TENAYA and LUCERNE) demonstrated the potential to extend the time of treatment with Faricimab at up to 16-week intervals between each injection with protracted efficacy in patients with nAMD.^1^ However, its effects on PCV are not clearly established yet.

Herein, we report a case of an unresponsive PCV that was successfully managed with Faricimab injections.

### Case description

A 56 years old female patient was referred to our clinic because of a large pigment epithelial detachment (PED) with persistent associated subretinal fluid (SRF) in her left eye (LE). The patient had previously undergone a pars plana vitrectomy and tissue plasminogen activator (tPA) injection in the right eye (RE) due to a subretinal haemorrhage associated with retinal pigment epithelium (RPE) hemorrhagic detachment, subsequently developing a disciform scar in that eye. The LE had been previously administered 3 Aflibercept (Eylea) intravitreal injections with no improvement.

On ophthalmic examination, Best-Corrected Visual Acuity (BCVA) was 20/200 in the RE and 20/32 in the LE, intraocular pressure was within normal limits and anterior segment examination resulted unremarkable.

**Figure 1.**
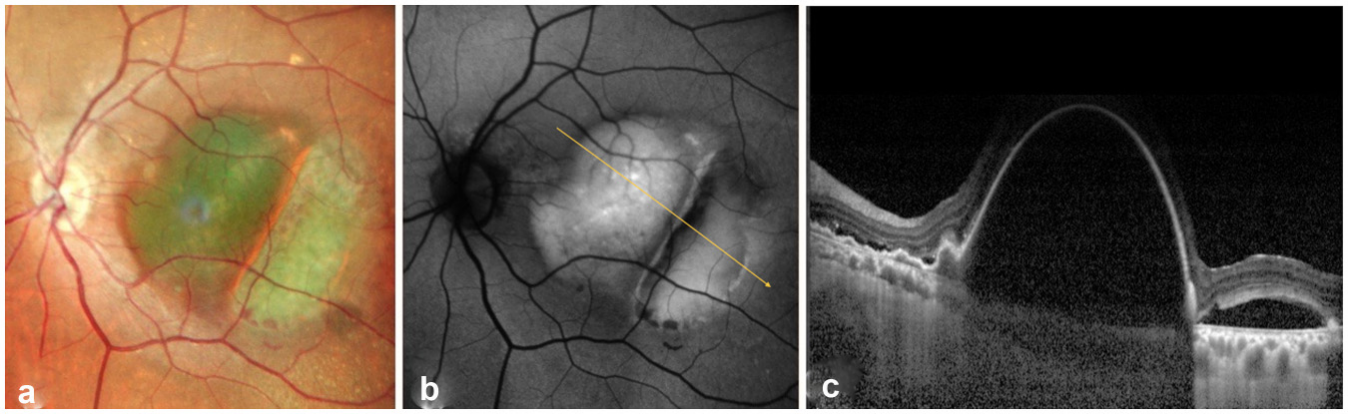
(a) Fundoscopy of the left eye, (b) Autofluorescence (AF) of the left eye, (c) Enhanced depth imaging mode optical coherence tomography (EDI-OCT) of the left eye.

Funduscopy of the RE showed a foveal disciform scar, whereas the LE presented a circular pigment epithelium elevation with an adjacent slighly more traslucent area in the fovea (Figure 1(a)).

Autofluorescence (AF) of the LE showed hyperautofluorescence in the foveal zone (Figure 1(b)) whereas OCT examination revealed a large subfoveal serous PED with associated subretinal fluid (Figure 1(c)).

**Figure 2.**
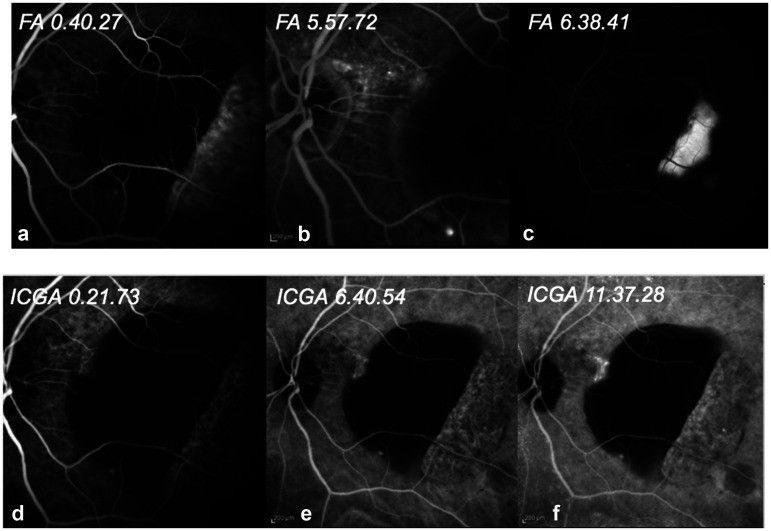
Showcase of different phases of fluorescein angiography (FA)/ indocyanine green angiography (ICGA). The retinal pigment epithelium (RPE) tear can be noticed in FA and ICGA temporally to the pigment epithelium detachment (PED).

Fluorescein angiography (FA) demonstrated blockage of the lesion throughout all phases with an adjacent area of pooling, correspondent to SRF accumulation. (Figure 2(a-b-c))

Indocyanine green angiography (ICGA) revealed an area of hyperfluorescence (leakage) better defined in the late phases localized nasally to the PED. (Figure 2(d-e-f)). A RPE tear resulted evident in FA and ICGA temporally to the PED.

OCT angiography revealed the full extension of the PED and confimed the RPE tear temporally to the fovea.

The patient was treated with a loading dose of 3 monthly intravitreal injections of Brolucizumab combined with PDT in the LE. In the follow up, a partial response to the therapy was noticed with reduction of the SRF but persistence of the PED and the VA reduced to 20/50 due to the RPE tear.

**Figure 3.**
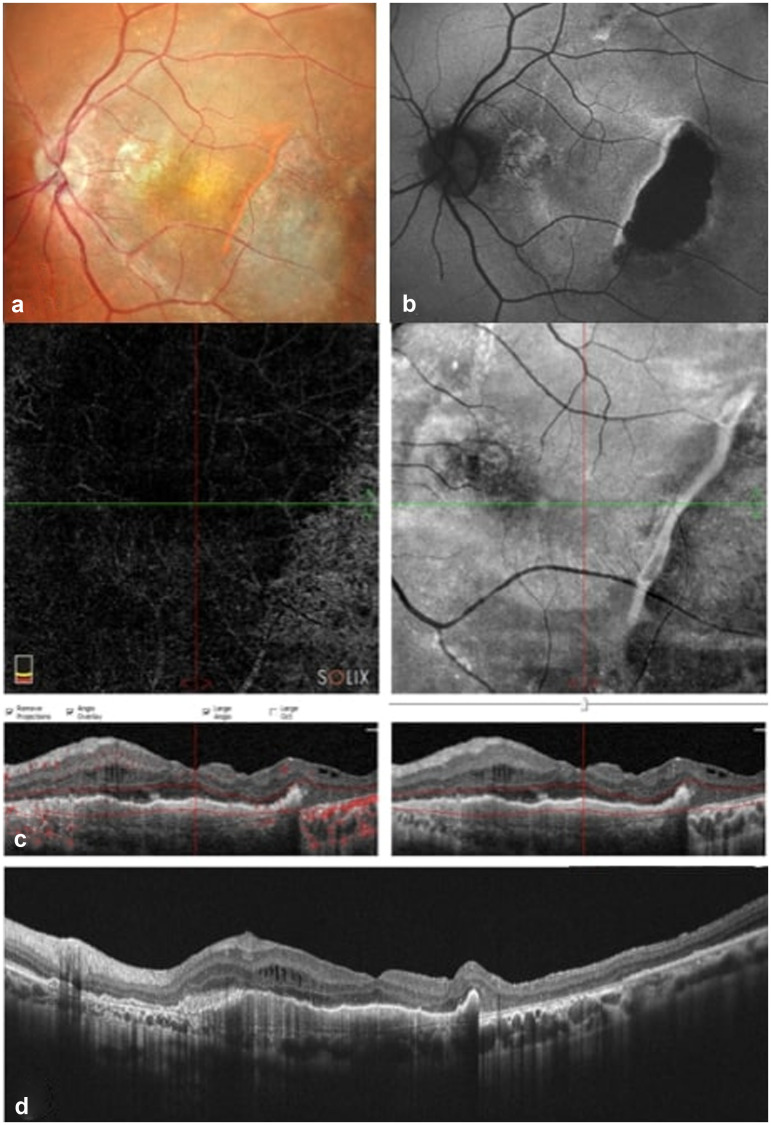
Imaging at 2-months follow-up post 4 Faricimab injections. (a) Fundoscopy, (b) Autofluorescence (AF) showcasing an hypoautofluorescent area correspondent to a dry atrophic area, (c) OCT- angiography and OCT en face (upper images) and OCT (down), (d) OCT displaying complete resolution of subretinal fluid (SRF) and size reduction of the pigment epithelium detachment (PED).

Subsequently, the patient underwent 4 monthly injections of Faricimab in the LE. On OCT complete resolution of SRF and size reduction of the PED was orbserved at the 2 months follow up. (Figure 3(c)) VA improvement to 20/25 was also noticed.

## Conclusions

According to previous epidemiological studies, PCV prevalence has raised to about 22–62% among nAMD cases in the Asian population and to about 10–20% in Caucasian population.^2^

PCV manifestations include neurosensory layer detachment, recurrent serosanguineous maculopathy, branching neovascular network, and aneurysmal neovascular lesions defined polyps. Compared to nAMD, eyes with PCV have a higher tendency of presenting with serous PED, massive subretinal hemorrhage and break-through vitreous hemorrhage.

Various treatment options have been recommended for PCV including PDT monotherapy with verteporin infusion, anti-VEGF monotherapy, and a combination therapy.

PDT uses its thrombotic property to enhance polyp regression and to reduce the exudation. Numerous studies have also demonstrated a benefecial effect with combination therapy compared to antiVEGF monotherapy in PCV treatment.^3^

Anti-VEGF therapy is considered first line treatment for PCV, even more so in cases where PDT cannot be performed due to significant hemorrhage or exudation preventing the visualization of polyps in ICGA.

Many Anti-VEGF agents have been studied in PCV treatment. Ranibizumab is a recombinant humanized monoclonal antibody and VEGF-A antagonist. The Everest study reported that PDT, either alone or in combination, resulted significantly more effective compared to ranibizumab monotherapy in obtaining complete regression of polyps (77.8% and 71.4% vs 28.6%).^3^

Aflibercept is a soluble decoy receptor that binds vascular endothelial growth factor-A (VEGF-A), vascular endothelial growth factor-B (VEGF-B) and placental growth factor (PIGF).

The Planet study proved that Aflibercept monotherapy was comparable to the combination therapy of Aflibercept/PDT in BCVA results with less than 15% of patients requiring rescue therapy with PDT.^4^

Brolucizumab is an anti VEGF-A humanized monoclonal single chain Fv antibody fragment. Compared to other anti-VEGF agents, Brolucizumab has been proven to be more effective in morphological and angiographic outcomes in PCV. The HAWK PCV subpopulation analysis described robust and sustained BCVA gains with Brolucizumab when compared to Aflibercept.^5^ Additionaly, higher polypoidal regression rates of about 78.9% were reported with 3-loading doses of Brolucizumab, compared to lower percentage of regression attained by Aflibercept or Ranibizumab monotherapy.^6^ Unfortunately, cases of occlusive vasculitis have presented following Brolucizumab injections.

In 2022 US-FDA-approved a new anti-VEGF agent Faricimab with an additional angiopoietin-2 inhibitory effect to treat wet AMD and Diabetic macular edema (DME). Angiopoietin-2 (Ang-2) expression has been proven to increase in the vascular endothelium in the pathologic phase of macular neovascularisation (MNV) in wet AMD (wAMD) thus inhibiting angiopoietin-1 (Ang- 1) from binding tyrosine kinase immunoglobulin-like receptors (Tie 2) on the surface of vascular endothelial cells and resulting in capillary inflammation or pericyte loss.

The inhibition of angiopoietin-2 most likely promotes vascular stability requiring less frequent injections than other anti-VEGF therapies. However, its efficacy and safety are not clearly established in PCV management yet.

In our case, the patient had shown poor response to previous treatment with Aflibercept monotherapy and combined Brolucizumab/ PDT therapy. Meanwhile, a loading dose of 4 Faricimab injections led to an improved BCVA, reduction in SRF and reduction in PED height. Interestingly, as observed in our case, a marked reduction in the PED height in PCV eyes with predominantly serous PED whose treatment was switched to Faricimab from other anti-VEGF agents was noticed in a recent study published in early 2024 by Ibrahim et al.^7^ According to Ibrahim et al results, Faricimab may have a higher beneficial effect on “sub-RPE fluid”

Moreover, the reduction in the PED height could be attributed to a reduction in leakage and reabsorption of fluid from anti-VEGF therapy, as well as a contraction of fibrocytes and other inflammatory cells.

In Ibrahim et al.'s study, choroidal thickess (CT) resulted also significantly reduced in the post-switch visit compared with the switch visit. Indeed, Angiopoietin 2 (ANG-2) levels have been reported as increased in eyes with pachychoroid features, suggesting a possible role of angiopoietins in choroidal remodelling.^8^

The inhibition of both ANG-2 and VEGF-A by Faricimab may promote choroidal vascular remodelling in patients with nAMD and a pachychoroid (such as PCV) showcasing a possible role of this new anti-VEGF drug in pachychoroid conditions.

In consonance with our case, a marked reduction or complete remission of fluid in Aflibercept-refractory PCV was observed in another recent study from Tamiya et al.^9^

In their paper about 27.3% of PCV eyes showed complete resolution of fluid two months after a single dose of Faricimab.

Furthermore, safety of loading therapy as well as visual and anatomical improvement with Faricimab in PCV eyes have been reported in a study published in 2023 by Mukai et al.^10^

The above-mentioned paper highlighted that the possible anti-inflammatory effects of ANG-2 inhibition contributed to the low rate of ocular infammation, confirming the safety of Faricimab injections. Additionaly, in line with our observations in their study polypoidal lesions in patients with PCV showed complete regression in 50% of the patients at the three months follow up, BCVA improvement, and mean subfoveal choroidal thickness reduction after receiving three consecutive monthly intravitreal injections of Faricimab.

Most notably, our case highlighted another aspect of this new antiVEGF drug: the superiority of Faricimab compared to combination therapy of antiVEGF and PDT in PCV. A loading dose of Faricimab achieved better results in BCVA improvement and SRF resultion when compared to Brolucimab/PDT combined treatment in PCV.

The increased levels of ANG-2 observed in pachychoroid-related disorders, combined with the efficacy of Faricimab in treating serous PED, suggest that baseline factors such as initial choroidal thickness and PED type may help predict a better response to Faricimab compared to other anti-VEGF agents. These factors could serve as biomarkers for identifying patients who are more likely to benefit from Faricimab, particularly in cases of refractory PCV. This aspect could assist clinicians in making more appropriate decisions when determining whether to switch treatments.

## Conclusions

Faricimab has shown to be an optimal alternative treatment in PCV cases refractory to other types of treatments. Determining the optimal timing for switching to Faricimab in refractory PCV cases is crucial, particularly given the potential for a superior response following the failure of other treatments, including combination therapy, as demonstrated in this case. The conclusive efficacy, durability, and safety for the use of Faricimab in treatment of PCV will be analyzed further in the SALWEEN study, which is currently enrolling.
